# How to Start a Fight: A Qualitative Video Analysis of the Trajectories Toward Violence Based on Phone-Camera Recorded Fights

**DOI:** 10.1007/s10746-022-09634-6

**Published:** 2022-10-10

**Authors:** Don Weenink, René Tuma, Marly van Bruchem

**Affiliations:** 1 University of Amsterdam, Amsterdam, Netherlands; 2 Technische Universität Berlin, Berlin, Germany; 3 Open University Netherlands, Heerlen, Netherlands

**Keywords:** Interpersonal violence, Video analysis, Ethnomethodology, Interactionism, Trajectory

## Abstract

We aim to contribute to recent situational approaches to the study of interpersonal violence by elaborating the concept of trajectories. Trajectories are communicative processes in which antagonists act upon each other’s bodily and verbal actions to project a direction for the interaction to take, which is then (con) tested in the exchanges that follow. We use the notion of trajectories to gain insight in how participants turn an antagonistic situation into a violent encounter, which we contrast to interactionist and micro-sociological understandings. Using ethnomethodological and conversation analytical tools, we detail the trajectories of three violent encounters, captured on phone camera recordings to answer the question how verbal and bodily exchanges project physical violence. Methodologically, our contribution shows how bodily actions can be studied in visual data. Our cases show how antagonists move the interaction toward violence by creating a metaconflict revolving around the conditions under which the interaction will become a physical confrontation; what we call the contested projection of violence. We conclude that the concept of trajectories offers a useful analytical tool to detail the shifts and turns of the interactive process—notably it’s bodily dimensions— that characterize antagonism and violence. Substantially, our analysis raises questions about conceptualizations of the emotional dynamics (notably the role of dominance) of violence, as proposed by earlier micro-sociological and interactionist work. We therefore suggest that future studies engage with these issues in more detail and in larger datasets.

## Introduction

In the past decade, interactionist and phenomenological approaches have provided new understandings of what happens in situations of interpersonal violence. Especially Collins’s (2008) micro-sociological theory has spurred research on the emotional dynamics of violence (Bramsen, 2018; Gross, 2016; Klusemann, 2012; Nassauer, 2019; Weenink, 2014). Ethnomethodological and conversation analytic studies (Elsey et al., 2018; Lloyd, 2017; Watson & Meehan, 2021; Whitehead et al., 2018) deployed refined situational analyses to produce insights into the situational contingencies and unfolding of violent encounters. Finally, recent phenomenological studies have inquired the lived experience of interpersonal violence, focusing on issues of intersubjectivity, embodiment and affective dimensions (Ciocan, 2019; Roehl & Kalthoff, 2013; Staudigl, 2007, 2013; for a similar position from a different approach Lindemann, 2015).^1^

Our contribution aims to advance these situationalist perspectives both theoretically and methodologically by considering interpersonal violence as a communicative process, a trajectory in which the antagonists use each other’s’ bodily movements, gestures, gazes, and verbal utterances to move the interaction toward (and away from) a physical confrontation. We use the notion of trajectories in granular analyses of video recordings of three incidents of “street violence” sourced from the internet to answer the question: How do people turn an antagonistic interaction into a violent encounter? More specifically, we consider how sequences of verbal and bodily exchanges project physical violence. As we will show, the paths toward violence are not unidirectional but are marked by turning points at which the interaction could have taken a different direction.

Our study is informed by ethnomethodologically oriented studies of violence (Elsey et al., 2018; Lloyd, 2017; Whitehead et al., 2018), however, we add ideas from other theoretical developments, such as communicative construction (Knoblauch, 2020) and symbolic interaction (see below). First, we consider similar violent incidents in public urban spaces as analyzed by Whitehead et al. (2018), but instead of focusing on how antagonists use “risk factors” as interactional resources, we study the entire interactional process as captured on the recording. Second, our analysis considers not only verbal utterances but also bodily modes of communication because our interest is in interpersonal violence as bodily action rather than in depersonalized forms of violence, as in the notions of structural and symbolic violence (Roehl & Kalthoff, 2013: 112). Third, our study is methodologically informed by Lloyd’s (2017) visual analysis of an incident between two mountain bikers. Adding to his work, we develop the notion of trajectories, aiming to provide a conceptual tool to study the emergence of violence. Finally, our work connects to interactionist insights into how encounters turn violent (Athens, 2005, 2015; Collins, 2008; Felson, 1982; Luckenbill, 1977). We add to this literature by showing that antagonists move the interaction toward violence by creating a metaconflict about the conditions under which the interaction will become a physical confrontation. We call this the contested projection of violence.

### Extant Interactionist Research on Interpersonal Violence

The interactionist tradition^2^ mostly views violent encounters as Goffmanian performances that revolve around maintaining “face”—a situational identity and a claim on how others should value and treat a person (Goffman, 1967). When antagonists perceive that the other has tried to project a negative identity onto them, for instance, by insulting, humiliating, or neglecting them, they may turn to (threats of) violence to “save face” (Goffman, 1967: 5–45 and later applications to violence: Felson & Tedeschi, 1993; Luckenbill, 1977; Fagan & Wilkinson, 1998; Polk, 1999). People may also engage in “character contests”—confrontations between opponents who do not give in (show character) as they try to save face at the other’s expense in sequences of provocations and challenges that can escalate into violence (Felson & Tedeschi, 1993: 109; Goffman, 1967: 217f.). Following this Goffmanian reasoning, interactionist scholars of violence have developed conceptual models of how violent encounters develop. Among the pioneers was Luckenbill’s (1977) study based on judicial case files. Luckenbill outlined the interactional steps leading up to violence: (1) one of the parties makes an opening move toward the other party, which is (2) interpreted as a personal affront by the other party so that (3) the situation becomes a contest about saving face, that might take the interactional form of a verbal challenge or ultimatum. (4) A “working agreement” is reached that violence is an appropriate means to settle the contest, in which both parties remain engaged in the contest rather than leaving the scene or apologizing. (5) Both parties engage in physical violence, and when (6) one party falls or is otherwise subdued, the situation ends. Athens’ (2005, 2015) elaboration of Luckenbill’s model—which posits dominance as the master motive driving violent encounters (see also Gould, 2003)—has five interactional steps comprising role claiming, rejection, sparring, enforcement, and determination. Following the centrality of dominance in his model, Athens does not agree that trajectories toward violence can comprise a “working agreement,” as Luckenbill would have it. However, both Athens’ and Luckenbill’s models leave unanswered the question why saving face or establishing dominance requires physical violence specifically and how the antagonists accomplish it.

While the work of Luckenbill and Athens postulates a priori “motivation” or situated goal of the actors, Randall Collins’s (2008) micro-sociological theory of violence refrains from motivational explanations. A key premise in his work is that most people do not easily commit violence because the confrontational tension and fear produced in antagonistic encounters forms a barrier. Collins proposes four ways in which people overcome this barrier. In two of them, tension and fear are superseded by a feeling of dominance stemming from situational asymmetries, for instance, when a group of onlookers supports one party or when one party remains passive or is otherwise perceived as weak.^3^ Interestingly, whereas Luckenbill’s and Athens’s models indicate that violence ends when one party attains dominance, it forms a condition for it to start in Collins’s model. We consider dominance as a situational asymmetry in which one party is no longer able or willing to pose a threat to the other.

Collins’s micro-sociological theory, and the availability of video data that allow to study the proposed emotional mechanisms, has boosted research that gets closer to violence than before, yielding insight in the situational-emotional conditions for violence to emerge (see Bramsen, 2018; Klusemann, 2012; Nassauer, 2019). However, these studies’ orientation on a-priori emotional processes prevented them from undertaking more detailed scrutinizing of the interactional processes in which these emotions are generated (see for a similar critique: Elsey et al., 2018: 316f.). As Bowman et al., (2018: 220; see also Whitehead et al., 2018: 330) noted, these studies have prioritized the emergence of more static “intraindividual” emotional features, rather than focusing on the moment-by-moment interactional, “interindividual,” processes. In this respect, it is telling that Collins (2008: 79 ff.), though referring to conversation analysis literature, primarily relies on photographs instead of video data, which stands to the identification of factors versus the analysis of action sequences. Whereas Nassauer (2019) uses video material, her work also prioritizes the identification of emotions and behaviours.

Following the leads of Lloyd (2017), Elsey et al. (2018), and Whitehead et al. (2018) we focus on how the antagonists themselves organize the trajectories toward and of violence. These studies’ use of video data is informed by the ethnomethodological and conversation analytical approach (see Sacks et al., 1973). Earlier work in this tradition, such as the study of ritual insults (Labov, 1972) and Goodwin’s (1987; 1991) research into sequences of accusations and responses, highlighted the details of linguistic turn-taking in antagonistic interactions. In our study, we attempt to identify trajectories as they are generated by participants’ bodily and verbal turn-taking.

The above mentioned ethnomethodologically informed studies have opened up a productive theoretical contrast to Collins’s micro-sociology of violence. Whereas an emotional impediment to violence is central in the former theory, Whitehead et al.’s (2018: 333, 336) analysis based on camera phone recordings showed that antagonists pushed the interaction toward violence, and/or felt warranted to do so based on their interpretation of the actions of the other.^4^ They also show how antagonists use provocative slurs and insults related to wider social divisions (pertaining to race, gender and class) to both prospectively and retrospectively account for the use of violence. Such provocations force the other to either leaving intense denigrations unanswered or confronting the opponent, escalating the situation further. Lloyd’s (2017) sequential analysis of a conflict between two cyclists highlights not only the verbal but explicitly also the bodily and spatial dimensions of the interaction. His analysis leads him to critique Collins’s theory for not giving enough attention to the sequential details of the interaction, notably concerning the interactive bodily intertwinement into the environment, which has been addressed by Katz (1999) in his study on road rage.

We aim to contribute to this debate by considering to what extent trajectories toward violence are characterized by situational asymmetries and the circumvention of emotional thresholds or rather by antagonists’ ongoing actions that push the interaction toward violence. Furthermore, we will consider the validity of Luckenbill’s and Athens versus Collins’s models with regard to the role of dominance in how fights start and end. However, our main contribution is to provide a conceptual and methodological approach to study interpersonal violence, which we use to show how participants move the interaction towards a fight. We enhance the ethnomethodological and conversation analytical approach with new developments in multimodal video analysis (see methods section), notions from symbolic interaction, most notably the idea of trajectories, as well as ideas taken from communicative constructivism (Knoblauch, 2020). Communicative constructivism highlights the role of knowledge to participate in communicative action, including the bodily and material aspects of it.

## Trajectories, Segments and Turning Points

We propose a conceptualization in terms of *trajectories* to understand the interactional contingencies of violence (based on Strauss, 2010 who developed the trajectory concept in his analysis of future orientedness in medical practices). However, we adapt the concept to micro situations. Whereas the term trajectory is usually read as predetermined and teleological, the meaning we give to it is similar to Gerson’s (1983) “work arc,” which captures both the mutual co-construction and the open-endedness of our understanding of trajectory. We prefer to use the term “trajectory” rather than “work arc” for the sake of generalizability but we highlight that trajectories are not unidirectional. In our usage, an interactional trajectory refers to the researchers’ reconstruction of how antagonists act upon each other’s actions to project a direction for the situation to take. Each action is also inherently a retrospective interpretation (account) of how the previous action was understood (Schegloff, 1968) and each action also projects a series of subsequent actions and prospective interpretations. In accordance with Copoeru (2020: 260), we thus view interpersonal violence as the outcome of “a dynamic embodied interaction” and we add that violence is such interaction itself. Trajectories thus capture communicative processes; they can be considered enactments of cultural knowledge of what a fight should look like. These projections are then tried and (con)tested in the exchanges that follow. The idea of “looming violence” captures the notion of projections aptly: antagonists (and the ones filming the event) have a sense of how the situation could evolve. And once “violence is in the air,” it becomes a point of orientation that directs the actions of the antagonists, either toward or away from its actualization. Following Ciocan (2020: 210f.) this moment forms “a special type of imminence” because it overwhelmingly imposes a fearful future on the experiential present. Thus, violence becomes analytically salient when the antagonists start to orient to its potentiality (see also Whitehead, 2012: 331). The notion of trajectory therefore also captures the specific temporality of violence, which is, as Copoeru (2020: 259–261) indicates, dynamically self-structuring; the dynamics itself are formed in the encounter, in the (violent) present. However, whereas Ciocan (2020: 206, 211) argues that violence is strongly present-oriented, our notion of trajectories is grounded on the idea that individuals’ (pre-) violent responses to one another are always projections of a future line of action.

Trajectories of violence are probably not unidirectional, with all actions oriented toward the looming prospect of a physical confrontation. They are likely characterized by what we call *segments* of varying intensity. Segments are parts of trajectories in which participants perform sequences of actions of a similar kind that are considered intelligible and relevant by the participants at that point in the trajectory (i.e., a segment in which antagonists scold each other or one in which they move closer to one another). *Turning points* mark a noticeable change in the type of actions; they introduce a segment of actions which orient the interaction more markedly toward physical violence or away from it.^5^ The entire trajectory therefore likely consists of intermittent pauses, withdrawal or distraction until there is a moment when actual physical violence becomes the dominant means of communication. Trajectories, segments and turning points are heuristic tools to grasp the complexity and open-endedness of the interactional process.

The term trajectory can be applied to various forms of violence; for instance, Reichertz (2020) uses the term to denote the more extensive (spanning longer time periods and larger numbers of people) and ritualized interactional steps toward football-related violence. One merit of using the notion of trajectories is that the analysis stays close to what the antagonists are doing when they engage in antagonism. Furthermore, we think the concept does justice to the future-orientedness of interactions (Tavory & Eliasoph, 2013) which becomes even more salient when violence lurks.

### Method

We analyzed three phone-camera video recordings of violent incidents which are part of a larger dataset comprising 131 clips obtained from freely available video uploading websites such as YouTube, Liveleaks and WorldStarHiphop, using search terms with the English keywords “assault,” “brawl,” “street fight,” and “violence” (see also Whitehead et al., 2018: 331).

This dataset was created for a larger research project on violence and de-escalation in urban public space (Weenink et al., 2022). The encounters we analyzed are commonly labelled as “street violence”. We used the following criteria to select the three cases. First, to delimit the scope of our study, we focused on encounters in which there were initially only two principal antagonists who used and/or were subjected to physical violence. Second, to observe the role of dominance in fights, we only included incidents in which, at one point in the interaction, one party used one-sided violence against a victim who no longer posed a threat. The theoretically relevant issue is then whether dominance paved the way for violence to occur (see Collins) or whether it ended the fight (see Luckenbill and Athens). Third, we selected cases which were different in terms of earlier theorizations. The first case exemplifies the character contests type of encounter which figures so prominently in the interactionist literature. The second case also qualifies as a character contest but here bodily gesturing plays a particularly prominent role in the trajectory, allowing us to highlight how bodily action can be studied as part of violent trajectories. The third case deviates from the character contest model because here one aggressive party one-sidedly had to put effort into construing the unwilling and nonaggressive opponent’s actions as a resource for developing a trajectory toward violence. Fourth and most importantly, we aimed for contrasting cases with regard to shifts in communicative modes as the encounter moved towards violence, in order to illuminate how our trajectory notion allows to understand this type of variation. The first case displays a distinct qualitative shift in the mode of communication when violence starts; there is a clear turning point which marks a progression from both verbal and bodily to only bodily actions. The second case does not show such a clear shift. Instead, participants are gradually moving toward a more and more non-verbal bodily form of communication as the encounter becomes increasingly violent. Finally, the third case is dissimilar to both the first and second in the sense that it does not show participants mutually shifting communicational modes at all. In this case of one-sided violence, non-verbal bodily actions gradually become more predominant in one participant’s communication, but not in his adversary’s communicative actions. In the words of Ciocan (2020: 199), violence is symmetrical in the first two cases and asymmetrical in our third case.

To identify trajectories, segments and turning points, we used procedures developed in ethnomethodological conversation analysis (Hutchby & Wooffitt, 1998: VI–VII; see Jefferson, 2004) and video analysis (Heath et al., 2010; Knoblauch et al., 2013; vom Lehn, 2019). Whereas earlier studies (such as Goodwin, 1991) have provided detailed conversation analytical accounts of the reciprocal taking up of the other’s verbal utterances in argumentative encounters, our analytical focus is on how participants move the trajectory forward through both bodily and verbal actions. We treated all visible and audible aspects of the situation that are recorded, such as gestures, facial expressions, and body movements, as potentially relevant to the analysis. Our task was then to reconstruct how these aspects gain meaning in the sequential context. Thus, our interpretations do not concentrate on the “what” but on the “how” of the production of actions (“Why this action now and in this particular way?”). Video analysis – even if grounded in reconstruction the participants’ accounts and using conversation analytic method—is always a basic hermeneutic enterprise; it starts with the aim to *understand* the actions of the participants, their reciprocal accounts and their sequential organisation (e.g., the meaning of specific gestures) using the researchers’ everyday embodied knowledge as a necessary resource (Knoblauch & Schnettler, 2012; see also Meier zu Verl & Tuma, 2020). To support the interpretative process, we used textual and visual representations (stills and drawings) to capture shifts in various communicative “modalities” (Kress, 2010) such as verbal utterances, gestures, body movements, more specifically the bodily-spatial communication of the participants, which was of pivotal importance to identify turning points. We used series of stills to capture these changes and embedded them into the transcript, a process we call transduction. We then created drawings from these stills, which forced us to focus on specific bodily gesturing and/or positioning. The drawings helped to highlight and show the organization of turning points performed by participants, such as the raising of the chin and the slight lifting of the body in case 1. Finally, drawings allowed us to create an overview of how people were positioned, which was relevant insofar as participants adjusted their actions toward that positioning (the circular formation of the audience in case 3). The transduction of the video into textual-visual representations was an iterative process of going back and forth between the video and the representations in progress. In the figures below, the drawings are related to the textual transcription, with the line numbers indicating the temporal position of the actions in the interaction.

Based on these procedures, we reconstructed turning points by matching transformations we observed in (a) the spoken language representing the verbal transcript; (b) the organization of turn-taking (such as rhythms of speech, pauses, overlappings); (c) bodily movements; and (d) bodily gestures and contacts. The conjuncture of several of those aspects indicated turning points, which mark the start of a new segment. The trajectories we found are based on the analysis of typical segments connected to one another through turning points. Our analysis did not focus on bystanders. However, the presence of an audience probably provides additional salience to the antagonists’ claims to be able to live up to their projections.

## Ethical Considerations

In our report of the analyses below, we use fictitious names for the antagonists for ease of understanding our argument. Antagonists used offensive language, mainly in the form of racial and gendered slurs. We discussed whether we should censor these utterances to avoid reproducing stereotypes (as was the case in the media attention generated when case 3 went viral). We decided to keep the original utterances because they are analytically significant; participants use them as communicative resources to move the trajectory into a certain direction (see Whitehead et al., 2018). For the same reason, we decided not to “decolor” our drawings of antagonists (see Figures) because their skin color was used as an interactional resource by the other party. To give an impression of the video material without revealing the identity of the persons captured on the recordings, we ‘cartoonized’ the clips we transcribed. The clips and our transcriptions can be viewed at https://osf.io/9j53w/.

### Case 1

The first case was selected because it exemplifies a relatively straightforward trajectory, allowing us to illuminate our analytical approach in detail. The incident took place in a subway train in a city in the U.S. While it lasts only half a minute, we observed five distinct segments. We focus on two antagonists, a dark-skinned male of Afro-American descent (“Shawn,” in the striped shirt) standing in front of his opponent, a light-skinned male sitting on a bench (“Reginald” wearing a red hat) (Fig. 1-1).
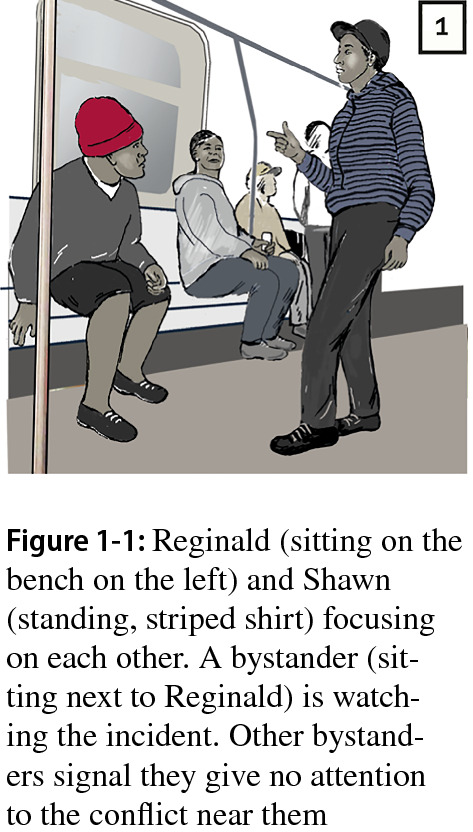


The first segment we observed lasted five seconds. At the beginning of the clip, Reginald projects a line of action that renders violence possible, provocatively saying, “Touch me”. While not explicitly communicated, Reginald draws on a repertoire of provocation, in which touch me means that the one who is hit first, is rightfully provided with an opportunity to strike back: if you touch me, I can hit you in self-defense. Shawn declines the offer based on his own projection of what will happen if he accepts the offer: “That won’t do you no good”. Both participants repeat these utterances several times, taking turns without interrupting each other. However, at each turn, hand gesturing (Fig. 1-2a/b) and the prosody of their utterances change. This indicates that the interactional sequences in this segment seem to be driven by each participant making variations in their own prior actions rather than using the other’s actions to ground their next move. The conflict seems stuck because the antagonists are not able to take the interaction toward a new, shared direction.
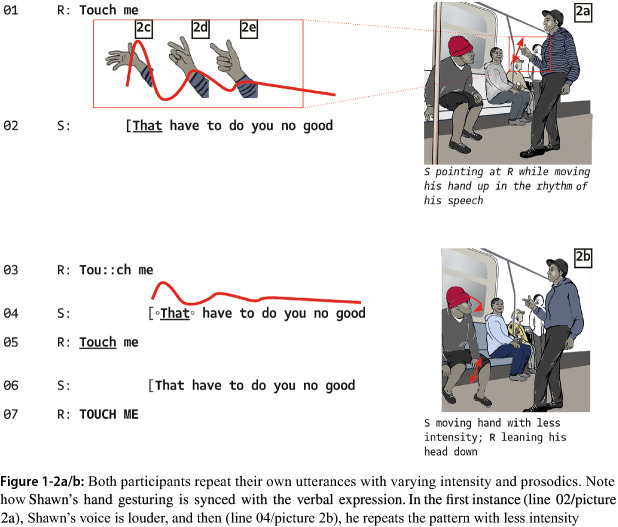


The start of a new segment is marked at the moment Shawn introduces his condition that projects physical violence (he might be using a prior unrecorded insult). Moving closer to Reginald, he proposes, “Call me a nigger again”. Reginald sticks to “touch me” as before, but turn-taking is now characterized by overlaps in utterances and proceeds with provocative insults and further threats (Reginald: “and I will fucking cut you”). However, at 11 s into the clip, we noticed a turning away from the potentiality of violence, which indicates to us the start of a third segment. Again, it is Shawn who reorients the trajectory. In response to Reginald’s threat, he takes a step back. Then he says, “Tough guy” (Fig. 1-3a/b). Instead of staying with the conditional proposal for action, Shawn chooses to classify, out of a universe of possible identifiers, his adversary as “tough”. The stepping away and the dismissive head movement (Fig. 1-3b) indicate to us that Shawn portrays Reginald as someone who is actually not so tough and that Shawn does not want to remain in this confrontation. Reginald reacts by raising his chin, moving his hand and slightly lifting his body to an upright position, thereby taking up more space, and in a more confrontational manner.
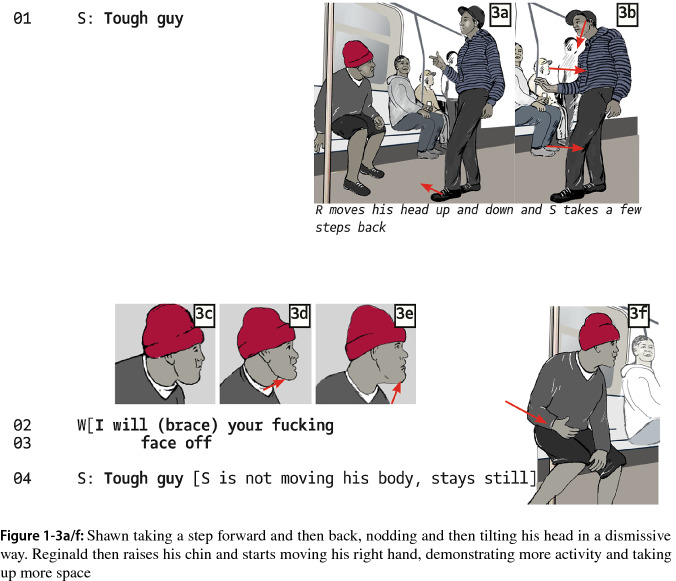


We argue the situation could have ended here because Shawn’s ambiguous “tough guy” and his stepping back offer a way out of the conflict. However, Reginald’s response to Shawn’s distancing brings the latter back into the conflict. This marks the start of a fourth segment. Reginald gets up slightly and moves his arms forward onto his knees, making more expressive bodily movements, which, given his sitting on the bench, can be seen as a more outspoken attempt to approach Shawn (Fig. 1-4a/b).
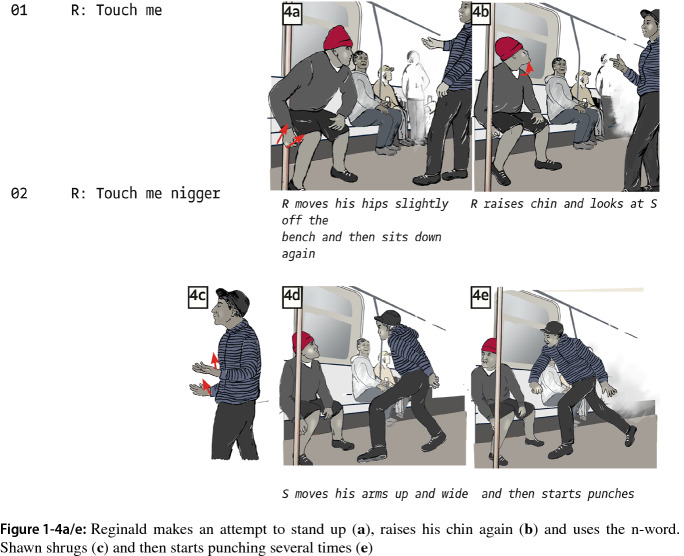


Reginald seems to prepare himself to act upon Shawn’s earlier condition for violence and provocatively raises his chin, uttering “touch me” again (Fig. 1-4a/b). Shawn replies by raising his arms in the air with palms open (shrugging), which to us displays puzzlement (Fig. 1-4c). Then, building on two prior interactional resources, Reginald repeats “touch me” for the ninth time and adds the n-word. It takes Shawn a short moment to realize what just happened. He moves slightly backward and then uses the space to charge forward, severely punching Reginald. After a series of blows, Shawn jumps back and utters “what” several times. Then after some more punching, he says, “I told you,” pointing his finger at Reginald in a similar way as he did earlier (see Fig. 1-1) three times until bystanders intervene.

At first sight, this case seems to follow Luckenbill’s and Athens’ interactional models; we can observe indications of a contest revolving around saving face (Luckenbill), as well as sparring, role claiming and rejection (Athens). We also find indications of Luckenbill’s “working agreement” about when to use violence, and the violence starts the moment the antagonists “agree” (saying the n-word). However, a closer look shows that “the working agreement” is actually a conflict. The antagonists develop a meta-conflict about what the conditions are under which violence can be used; which line should be crossed. While violence is not a necessary outcome of the interaction, the antagonists contest each other’s projected lines toward a physical confrontation. Furthermore, Shawn’s violence is one-sided and he continues to punch Reginald after he is already subdued, whereas Luckenbill’s and Athens’ suggest proportionality; in their models, violence is instrumental to decide who is dominant. Furthermore, we did not observe signs of Collins’s confrontational tension and fear in the antagonists. While the confrontation turned stuck for a moment and Shawn was about to withdrawn from the encounter at some point (his stepping back), this was not due to emotional inhibitions but to the antagonists’ inability to find a way to move the confrontation further. Moreover, a shift toward dominance, in the sense of a situational asymmetry in which one party no longer poses a threat to the other, did not precede violence, as Collins’ theory indicates. Instead, Reginald saying the n-word, his final “acceptance” of Shawn’s condition for violence, precipitated the latter’s assault. Moreover, throughout the confrontation, Shawn was in a much better position to attack then Reginald, but the asymmetry was apparently not sufficient to make the former turn to violence.

### Case 2

In this, case, two men from the U.S. had an argument in a McDonalds restaurant at a Chinese airport. Bodily actions figure more prominently as interactional resources in this case, enabling us to demonstrate how bodily forms of communication can be studied using our approach. Our identification of segments is therefore mainly based on bodily transformations (also because it was not possible to completely understand all verbal utterances due to the limited recording quality and the noisy surroundings). We focus again on the two main antagonists. The two are Gary (older white man wearing glasses) and Will (younger white man wearing white shorts). The clip starts with the antagonists standing very close to each other. Gary indicates that his age does not prevent him from giving his opponent a beating: “[I’m] 50 fucking years old, but I’m fucking your ass (up?)”) (Fig. 2-1a). While Gary aggresses, pushing his chest forward and coming closer to Will, the latter holds up his arms, a gesture we interpret as unwillingness to fight (Fig. 2-1b). However, Gary continues pushing forward and sternly faces Will, who then puts his flat hand with fingers stretched onto Gary´s chest, gently pushing him away while saying “bro” several times in an appeasing manner, while Gary threats to “fuck you up”. After pushing Gary aside, Will presents a smile that can be read as disdainful. There is ambiguity in Will’s smiling and touching of Gary’s chest; on the one hand, they seem to signal “nonaggressiveness”; on the other hand, these actions are understood as disrespectful by Gary, providing him with a reason to further the escalation.
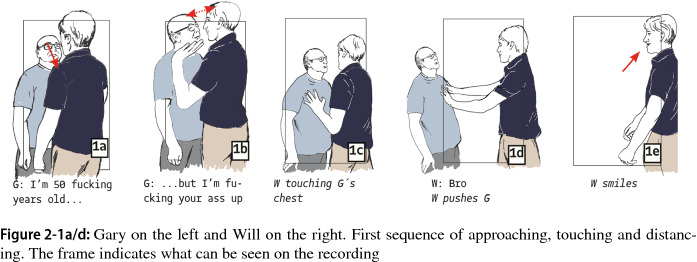


The approaching-touching-distancing pattern provides the main interactional resource for the segments to follow. For Gary to turn his projection “to fuck [Will] up” into bodily action, he needs Will to act upon the encroachment of his body space. In the ensuing segments, we observe an increased intensity of approaching-touching-distancing movements, with Gary initiating subsequent rounds of the pattern. Will, on his part, contributes to the eventual accomplishment of Gary’s projection. By holding his ground rather than leaving, he continues to provide Gary with the opportunity to encroach his body space. Will’s body thus provides an interactional resource to continue antagonism.

In the next segment we identified, Gary approaches again, but now Will is prepared and directly puts his hand onto Gary’s chest (Fig. 2-2a), pushing him away with slightly increased intensity (Fig. 2-2b). Moreover, he keeps his arms stretched out as a barrier preventing Gary from coming closer. The latter again utters threats to fight (“I gonna, I gotta getta”) and insults Will for not fighting him (“you wanna fucking be a bitch”) (Fig. 2-2c). From that moment, both parties are distanced again and argue (as we infer from their gesturing) for a moment (Fig. 2-2d).
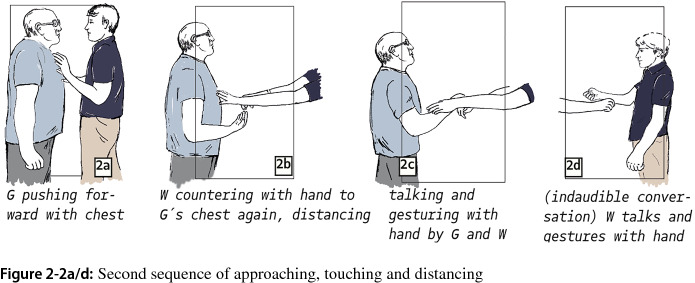


This pattern is repeated with increased intensity in the next segments (Fig. 2-3a/d), and we identify a turning point when Gary elaborates the touching of prior segments into grabbing (Fig. 2-4a/c). Now, the movement pattern has become approaching-grabbing-distancing, which is repeated two times with increasing intensity (Fig. 2-4a/c, 5a/c, 6a/c). The grabbing provides Gary with an opportunity to elicit a more aggressive reaction from Will. As Gary holds Will’s arm, he provokes the latter to free it with force (Fig. 2-6a/c).
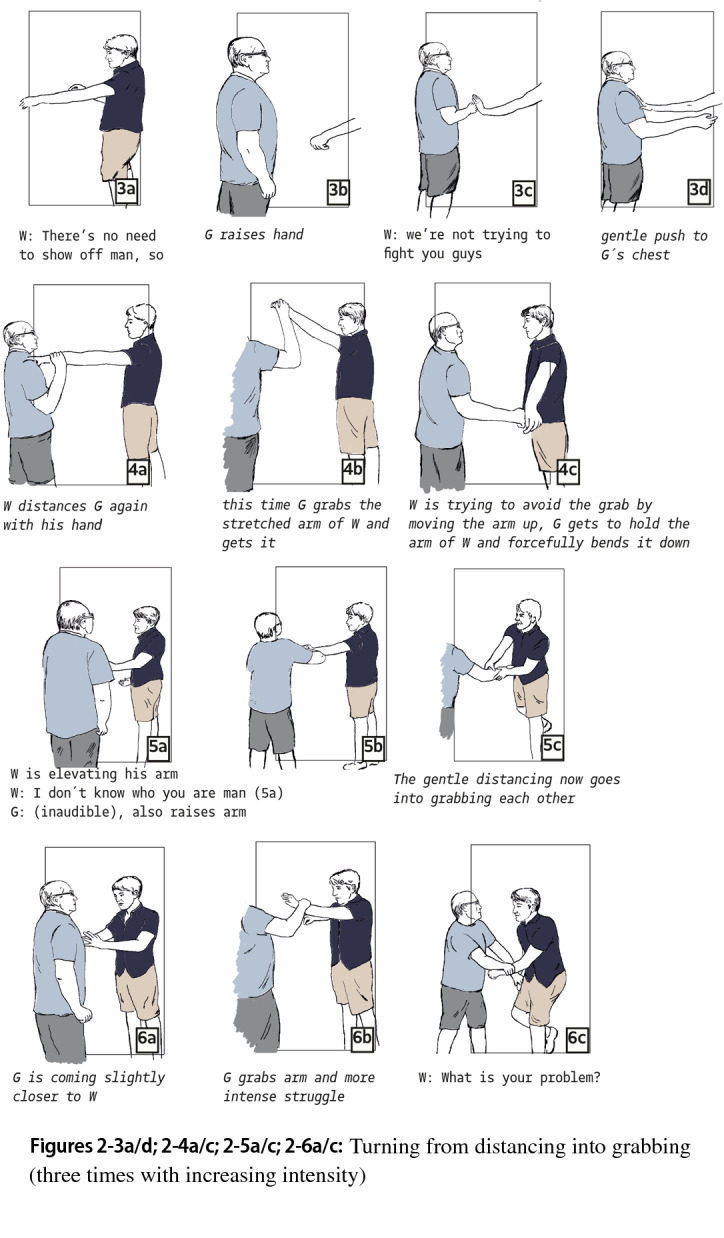


The next segment starts when Gary pushes forward and “charges” at Will with the full weight of his body (Fig. 2-7a/b). Whereas in the former segments bodily confrontations were followed by momentary retreats in which both parties stepped back and exchanged verbal utterances, we now observe continuous bodily engagement without talking (Fig. 2-7c; 2-8a). From this point, each action of the other is used to gain advantage in the bodily struggle. Thus, as Will turns to the side and tries to avoid Gary grabbing his neck, he loses his balance. Gary seizes the opportunity and throws Will against the wall (Fig. 2-8b, 2-9a).
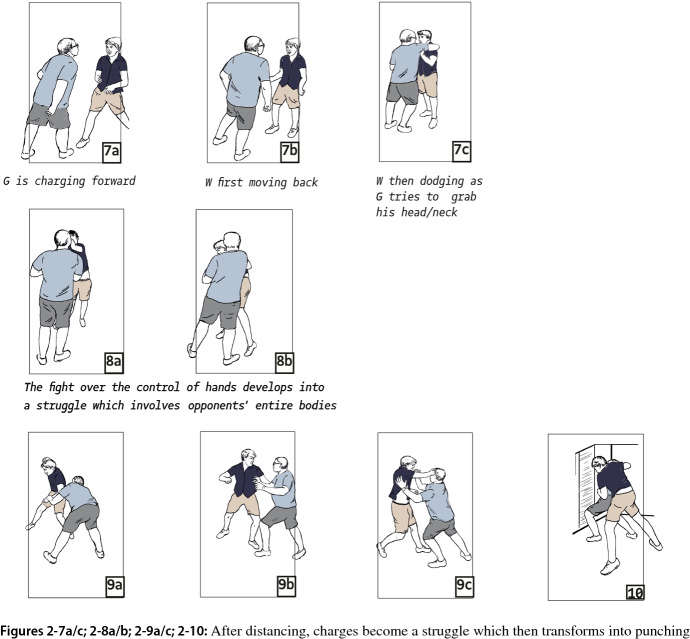


A last transformation can be identified, when Will, for the first time, introduces a new line of action. He changes the mode of the physical confrontation from grabbing and pushing to punching. In response to Gary’s pushing, Will punches Gary’s head, hitting Gary’s glasses, which fly away (Fig. 2-9b/c). As Gary stumbles, Will pushes him into a corner. Gary bends forward, with his head close to the ground. Will, who now has unhampered opportunity to strike, seizes the opportunity and unleashes a series of blows on Gary, who is in a vulnerable position (Fig. 2-10).

In this case as well, important elements of Luckenbill’s and Athens’ interactional models appear; the case could be interpreted in terms of a dominance contest, including a bodily version of Athens’ role claiming, rejection and sparring as Gary probes Will’s “line” by repeatedly encroaching the latter’s body space. And whereas Will displayed unwillingness to participate in escalation at the beginning, he stood his ground, thus providing Gary with the interactional resource to continue the antagonism and gradually increase the intensity of bodily contact. In this case, there is not a clear-cut line that demarcates the start of a physical confrontation. Rather, there is ambiguity; the antagonists disagree about whether and when the situation should be understood as a fight. They thus develop a new conflict that revolves around the conditions under which two-sided violent engagement starts. For his part, Gary provokes Will, using series of approaching-touching-distancing and then approaching-grabbing-distancing moves (Fig. 2-3/6). And once Will eventually engages in a physical, non-verbal confrontation, he introduces a new modality of violent action, from wrestling to punching (Fig. 2-9a/b; 2-10). So, in this case as well, the projected lines of action toward and of physical violence are contested. Moreover, as in the first case, violence is not just a means to decide who is dominant: when Gary is in a subdued position, Will’s violence continues and in fact becomes more fierce. Furthermore, Will’s reluctance to answer Gary’s proposals to engage does not seem to originate from confrontational tension and fear. Will stands steady, and shows signs of indignation and contempt rather than being tense or fearful. And whereas in Collins’s theory situational asymmetry is a precondition for violence to start, we observe that once Gary is subdued, the roles of the antagonists reverse: now Will uses one-sided and in fact more fierceful violence.

### Case 3

Whereas the two prior cases show how the participants provide interactional resources for the other to move the trajectory toward a projected violent outcome, this result is attained much more one-sidedly in the following case. In this incident, a white Dutch boy (“Tobi”) had agreed to fight someone (depicted in Fig. 3-1 as “original opponent”). The video footage starts at the moment Tobi indicates that fight is over due to his bleeding broken nose (according to documentation found on the internet). However, “Ben,” a Dutch boy of minority descent (not visible in Fig. 3-1, but see Fig. 3-4) then attempts to project a continuation of violence against the now unwilling and nonaggressive Tobi. In this more complex trajectory, the groups present form an audience which plays a crucial part; therefore we pay attention to group member’s actions, notably their bodily positioning and intermittent comments. The clip lasts 5 min, and it takes approximately 4 min until collective violence starts. We discuss a selection of the 18 segments we identified. They show how the group, lead by Ben, turns Tobi from an unwilling opponent into an offender.

At the beginning of the recording, Tobi sits on his knees on the ground. Group members, assembled in a circle around the victim (see Fig. 3-1), cheer that he is not done yet and that he must continue fighting. The spatial formation allows the youth to focus on the spectacle, a.o. through the use of phone cameras.
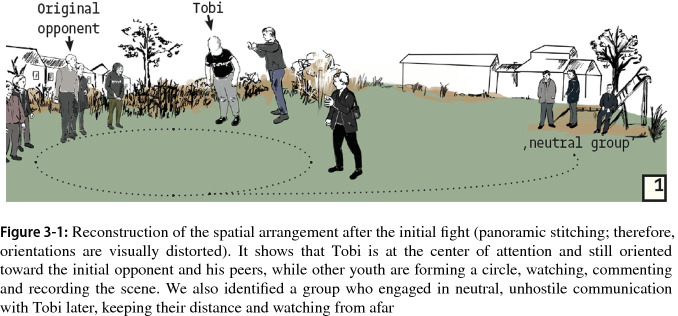


Tobi does not respond verbally. As he gets up from the ground, he draws attention to his body, wipes the blood from his nose, shakes it from his hands and spits. This bodily posturing is signaling that he is unwilling, or even unable, to continue (Fig. 3-2).
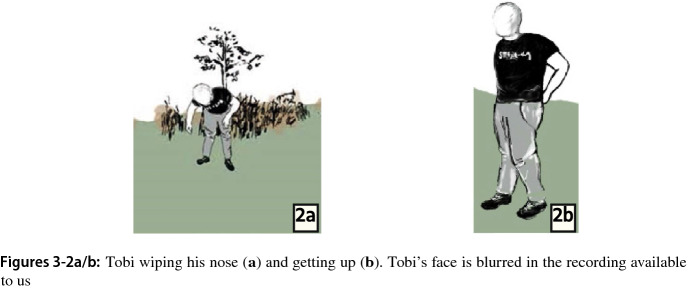


This segment already shows the main resources that will used to subsequently turn Tobi into someone who transgresses a norm, an “offender”: his refusal to continue the fight. After an intermittent segment in which Tobi walks away from the group, we identified the beginning of a new segment when one group member returns to the earlier theme that the fight is not over yet. This is taken up by other group members, who interrupt each other energetically and agree that the fight is not over and that Tobi should stay (see Fig. 3-3 Transcript). Their overlapping comments create a sphere of excited expectation for a fight to come. 
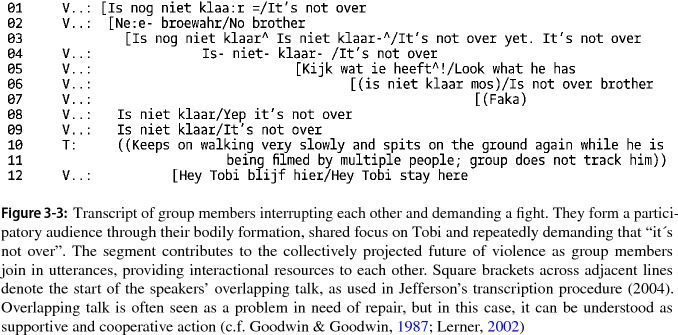


A next shift can be identified when Tobi responds to the group; he stops walking away slowly, turns around and looks toward the group, his body posture straighter now. He says that the fight is over because of his hurt nose. His remark introduces material to revive antagonism, which is immediately taken up by Ben. Ben loudly announces the line of action to move the situation toward a physical confrontation: “come over here, or I will fight you”. Tobi and Ben engage in argumentative turn-taking, with Tobi explaining why he cannot fight (due to his hurt nose). Ben’s gesturing becomes more intense and his voice becomes louder in this segment. When he says he “did not come to watch a 30-s fight,” he not only defines the situation as an entertaining spectacle but also denotes Tobi as blameworthy for spoiling his time. The group then realigns with Ben and Tobi, focusing their attention on their antagonism, while group members say they “haven’t seen shit,” legitimizing Ben to continue his efforts.

When Tobi does not respond, Ben picks up the prior theme of humiliation as a resource, saying Tobi is a “pussy” and “weak”. Ben thus portrays Tobi as “unworthy” to provoke a counteraction. The asymmetry between Tobi and Ben increases in this segment as Tobi returns to his earlier closed-in, inward-oriented bodily posturing while Ben adopts aggrandizing posturing and seeks support from the audience, which has formed a loose line between Ben and Tobi and the “neutral” group (we observed one of them inquiring about Tobi’s condition) at the playground in the back. As he humiliates Tobi, Ben tries to connect to the group several times. By pointing and “speeching,” he turns the conflict into a collective play. However, the group is not entirely focused, with group members having their own conversation about recording what is going on (Fig. 3-4). Ben addresses the audience when he gestures ostensibly toward Tobi, urging him to fight again (Fig. 3-5b). Ben is supported by a younger group member who steps forward and adds denigration as a new form of humiliating Tobi (Fig. 3-5c).
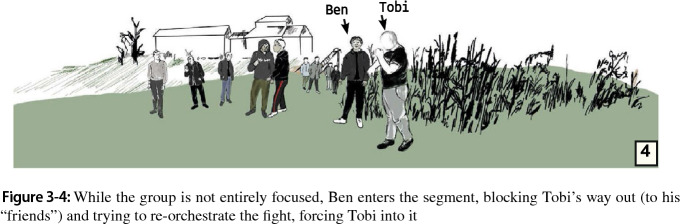

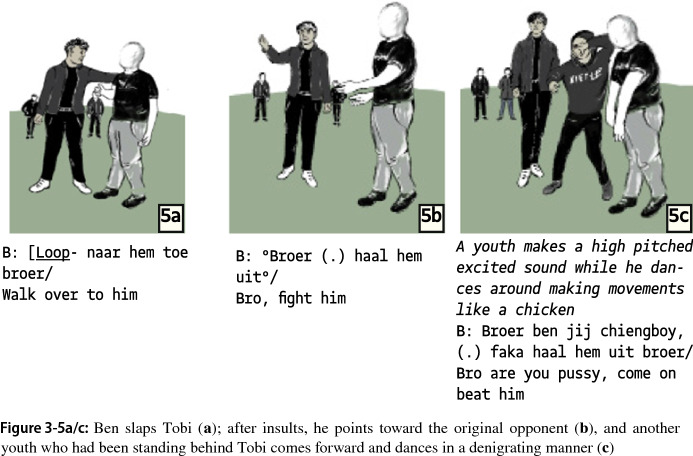


The asymmetry between Ben and Tobi is even more apparent when Ben switches from humiliation to a compelling projection of physical violence: he counts down, stating that he will use violence when the count is over. In response, Tobi again states that the fight is over. As the opponents reintroduce the earlier topic of disagreement, Ben becomes more angry. They start a dialogue in which they repeat their statements with variations, Tobi saying the fight is over, Ben saying it’s not (see also case 1 for repeated but varying statements as calling out reactions in one’s body).

A marked shift occurs when the argumentative turn-taking is replaced by one-sided humiliation. Ben slaps Tobi and explicitly remarks that he slaps him with his flat hand and, to emphasize the humiliation, adds that his slapping indicates “disrespect” (Fig. 3-6).
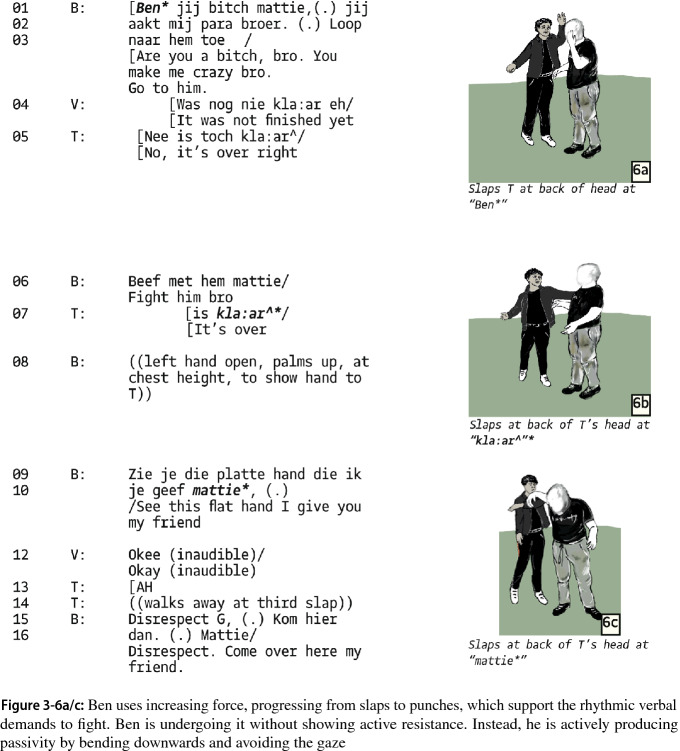


The following segments again show increasing asymmetry: as Tobi tries to get away, Ben and another group member follow him, while Ben increases the intensity of violence from slapping to punching which is accompanied by more slurs and verbal abuse (Fig. 3-7b/c). Tobi no longer tries to get away. He now stands in the bushes, bending over and minimizing. Ben is again demanding to “fight with him now”. The more passive Tobi is, the more work Ben has to put into move the interaction further. One way to do so is to construct the ensuing aggression as a punishment.
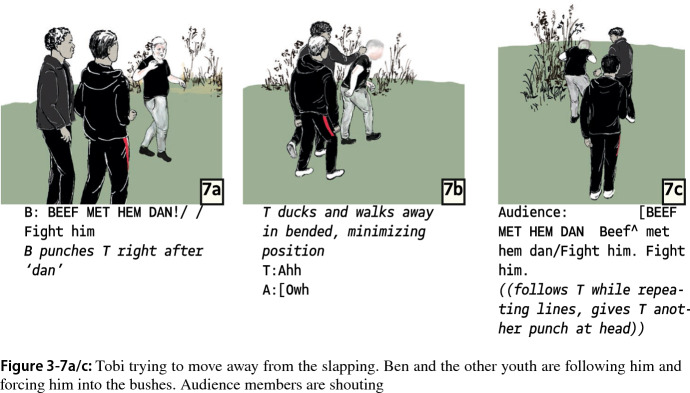


As Ben develops increasingly severe and painful violent actions, from slapping to striking at the back of Tobi’s head, he punctuates each violent act with exclamations, indicating that Tobi did something wrong: “I am sick and tired of this fucking shit of yours” (Fig. 3-8). Here, Ben might refer to Tobi’s earlier plans to fight a group member; he previously stated that he “didn’t come here to watch a 30-s fight”.
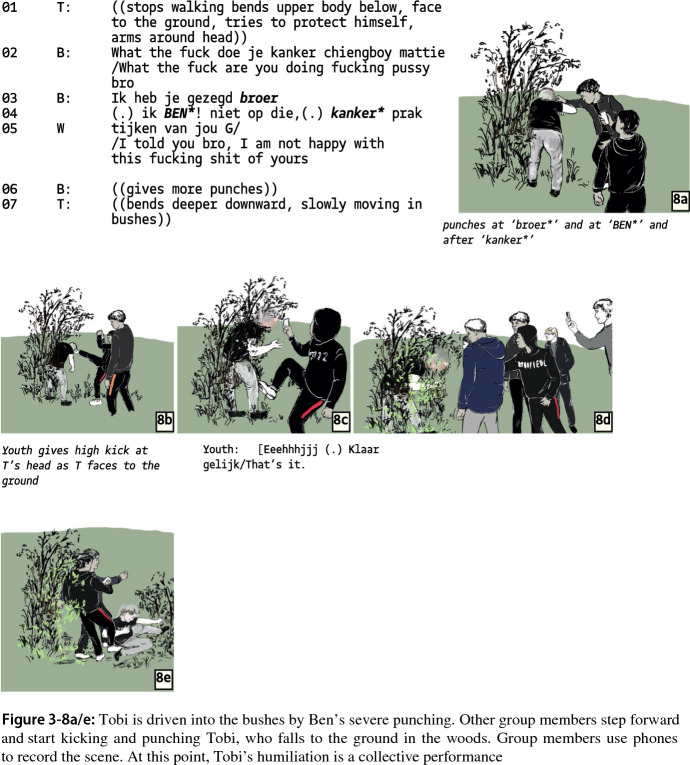


Ben has transformed Tobi from an unworthy opponent into an object of humiliation and punishment. While group members respected some distance from Tobi’s body space in earlier segments (Fig. 3-1 and 3-4), they now follow Ben’s example and kick Tobi (Fig. 3-8b). When Tobi falls on the ground in the bushes, he is followed by Ben and another group member, who start to kick him fiercely on his back and head while group members cheer and shout with excitement. Two other group members also kick Tobi on his back, although less fiercely (Fig. 3-8e).

To conclude, this case does not seem to fit so easily to the interactional models provided by Luckenbill and Athens. The most important element, a contest that revolves around attaining dominance, is missing. Instead, Tobi is reluctant to engage in a contest and does not propose conditions for starting a physical confrontation, which also means this case lacks a “working agreement”. Tobi’s reluctance creates an interactional problem for Ben: how to start a fight with an increasingly passive “opponent”. Eventually, Ben does not succeed in creating a fight. Instead, he performs “badness”; he demonstrates his ability to play a mean, one-sided humiliating game with Tobi with support of the audience (see Katz, 1988; Weenink, 2015). As in the other cases, the notion of a building up of confrontational tension and fear does not seem to fit very well to Ben’s unleashing of violence. We view his actions as establishing a trajectory that constructs a legitimate –in the sense of being supported by the audience– cause for violence. Also, even though situational asymmetry is clearly visible in the submission and passivity of Tobi, it does not immediately translate into violence. Rather, we observed a contested projection of violence, and this metaconflict –which is the only conflict in this case– is worked out by defining Tobi as an object of humiliation to toy with.

### Discussion

From the viewpoint of the antagonists, when and how violence starts is an interactional problem they must solve. The notion of trajectory is not only a descriptive category but also entails the participants’ knowledge and their “framing” of the situation, which is enacted by projecting a shared future; a potentiality that can be acted upon – contested, accepted or otherwise – by the participants. Therefore, trajectories are communicative interactions that (re)produce shared cultural knowledge about what a “fight usually looks like”. Transitions from “nonviolence” to “violence” and vice versa consist of forms of physical contact that require interpretation. A "knock out" blow might be a clear result, but also the pushing away or grabbing of the other’s hand is produced as indicative of at least a small struggle. In that sense, the trajectories we described are a mutual accomplishment of disagreement but far from an agreement to disagree. The three cases revolved around a meta-conflict about how to start a fight; the antagonists contested each other’s projections toward a physical confrontation.

However, our findings pertain to three cases only and it is not our intention to make generalizing statements about the wider population of violent encounters. Instead, we aimed to develop the notion of trajectories to advance earlier contributions by Luckenbill and Athens that present rather straightforward idealizations of violent interactions. Thus, based on thorough and detailed transduction of the video material, we could show the role that meta-conflicts play as precursors to violence, which has not been described in the literature before. Understanding violence as a trajectory also avoids a binary distinction between “nonviolence” and “violence” and allows to study the processual character of the phenomenon itself (see Bowman et al., 2018; Trotha, 1997).

Doing conflict and doing violence require shared bodily know-how of action sequences. This pertains not only to actual fighting techniques but also to provocative finger pointing, choreographies of evasion and pretending to be able to throw a punch (see also Schindler, 2011; Staack, 2019 for bodily knowledge related to fighting). Moreover, interpersonal violence is bodily action. The statement is obvious, but few studies have taken it into serious consideration (but see phenomenological inquiries into violence: Staudigl, 2007: 236). Wacquant’s (1995) study of learning how to box reveals the experiences of boxers managing and disciplining their bodies. However, Wacquant does not discuss how bodily action works in the interactional sequences that constitute violence. We hope to have shown that our methodological approach allows to analyze bodily actions (rather than “the body” or “embodiment”) as part of the communicative co-creation of violent trajectories: turning points are created by changes in bodily comportment, gesturing, and movements as well as by changes in speech and prosody.

Although we should be prudent to draw substantial theoretical conclusions based on our small dataset, our study begs questions about the role that dominance and confrontational tension and fear play in violent encounters. First, we could neither find indications that dominance is a precondition for violence (as in Collins’s theory) nor an end to it (as in Luckenbill’s and Athens’s models). Second, in line with Whitehead et al. (2018), we could not observe signs of a building up of confrontational tension and fear, which is seen as a crucial part of the emotional dynamics in face-to-face violent encounters in Collins’s theory. These questions clearly require more focused research, based on a larger dataset.

The notion of trajectories also allows considering what Ciocan (2020: 197) has termed the “various *modifications* of the experiential dimensions” in the sense of the shifts and turns that can occur in the sequence of (pre-)violent actions (see also Copoeru, 2020). Our notion of trajectories of violence allows to question a widely accepted idea about the experience of violence. Many studies assume that violence takes the form of a sudden “outbreak” or “outburst” of uncontrollable emotions (Ciocan, 2020: 199, 202–204; Collins, 2008: 83ff.; Roehl & Kalthoff, 2013: 112). It is true that violence emerges rapidly, but that does not mean it comes sudden, nor that the behavior of individuals is entirely shaped by their emotions. The notion of trajectory can help to study how violence itself comprises longer or shorter sequences of actions in which people interactively move the interaction toward or away to more or less emotionally intense forms of violence.

We close this paper by discussing a number of limitations of our study which need further elaboration in future research. First, our material leaves the why of interpersonal violence largely unanswered. As recorders often start filming when the conflict is already underway, we cannot provide an understanding of the origins of the antagonism. Data that allows to observe participants before the conflict (for instance, extended footage) would make it possible to use the notion of trajectory to show how participants create a conflict as a interactional accomplishment. Also, with regard to the why of violence, one could object against video data more generally that it does not provide insight in the subjective motivations of participants. However, whatever motivations individuals may have, interpersonal conflicts only materialize when they engage in engaging in antagonistic turn-taking. Phrased in our terminology, for motivations to become part of social reality, individuals need to project a trajectory. A further objection against video data is that it lacks contextualization; from video material alone, it is impossible to know how historically developed social cleavages, moral meanings and social relationships enter a conflict situation. Adding ethnographic components (Knoblauch et al., 2013) to video research, for instance, by using video elicitation techniques to contextualize the recorded situation would generally be the preferred method to counter this limitation.

Third, our three cases all ended in violence. Whereas we claim that the trajectories we described are contingent and not teleological –as we identified various turning points away and toward violence–, future studies could detail the trajectories of similar conflicts that did not end in violence to trace the particularities (in terms of turning points for instance) of non-violent and violent conflicts. More specifically, it is probably worthwhile to consider the transition to a bodily-only mode of communication in this respect, as our analysis of the second case shows.

Fourth, whereas we noted the role of the audience in our third case, a more systematic conceptualization of the role of third parties in trajectories of violence is needed, as studies point to their importance in dampening and also encouraging violence (Levine et al., 2011; Philpot 2020; Weenink et al., 2022). Empirical studies could take up the question of how open various trajectories are to intervention by third parties (see Coenen & Tuma, 2022).

Finally, we focused on a specific form of violent interpersonal encounters in public space, especially public forms that are explicit, visible and situationally bound, ephemeral encounters. However, other forms of violence, embedded in more durable social relationships and organizational structures could be conceptually understood as comprising multilayered trajectories, with various temporal horizons, situational and longer lasting. We are confident that the detailed study of sequential unfolding, the attention to bodily actions and the use of the non-determining trajectory concept can, however, provide insights into other forms of violence as well, even if video-based studies exhibit a certain bias towards visible violence.

## Footnotes

1 The literature on violence is vast. We focus on the micro-sociological and interactionist traditions, and make connections to recent phenomenological studies published in this journal.

2 This tradition goes back to Mead, who used the dog fight as an example of communication: “To illustrate this further let us go back to the conversation of gestures in the dog-fight. There the stimulus which one dog gets from the other is to a response which is different from the response of the stimulating form. One dog is attacking the other, and is ready to spring at the other dog's throat; the reply on the part of the second dog is to change its position, perhaps to spring at the throat of the first dog. There is a conversation of gestures, a reciprocal shifting of the dogs' positions and attitudes” (Mead and Morris 1974: 63).

3 In the other pathways, assailants avoid mutual antagonistic involvement. This happens when victims are attacked by surprise or stealth, such as when assailants approach their opponents from behind or afar. This article does not consider such forms of violence.

4 Their study refines Luckenbill’s (1977) earlier idea that violent encounters are characterized by a “working agreement” in which the antagonists continue to participate in the interaction when violence is looming.

5 Glasers and Strauss’ (1968: 6f.) notion of „crititcal junctures “ is different from our idea of turning points. His usage of the concept is similar to the models that Athens and Luckenbill propose. Our use of turning points is a more open and not predetermined heuristic tool.
